# The state of the antivaccine movement in the United States: A focused examination of nonmedical exemptions in states and counties

**DOI:** 10.1371/journal.pmed.1002578

**Published:** 2018-06-12

**Authors:** Jacqueline K. Olive, Peter J. Hotez, Ashish Damania, Melissa S. Nolan

**Affiliations:** 1 Departments of Pediatrics and Molecular Virology and Microbiology, National School of Tropical Medicine, Baylor College of Medicine, Houston, Texas, United States of America; 2 Texas Children’s Hospital Center for Vaccine Development, Baylor College of Medicine, Houston, Texas, United States of America; 3 Department of Biology, Baylor University, Waco, Texas, United States of America; 4 James A. Baker Institute for Public Policy, Rice University, Houston, Texas, United States of America; 5 Scowcroft Institute of International Affairs, Bush School of Government and Public Policy, Texas A&M University, College Station, Texas, United States of America

## Abstract

In a Policy Forum, Peter Hotez and colleagues discuss vaccination exemptions in US states and possible consequences for infectious disease outbreaks.

**Summary points**

- A social movement of public health vaccine opposition has been growing in the United States in recent years; subsequently, measles outbreaks have also increased.
- Since 2009, the number of “philosophical-belief” vaccine nonmedical exemptions (NMEs) has risen in 12 of the 18 states that currently allow this policy: Arkansas (AR), Arizona (AZ), Idaho (ID), Maine (ME), Minnesota (MN), North Dakota (ND), Ohio (OH), Oklahoma (OK), Oregon (OR), Pennsylvania (PA), Texas (TX), and Utah (UT).
- Several US “hotspot” metropolitan areas stand out for their very large numbers of NMEs. They include Seattle, WA, Spokane, WA, and Portland, OR in the Northwest; Phoenix, AZ, Salt Lake City, UT, Provo, UT, Houston, TX, Fort Worth, TX, Plano, TX, and Austin, TX in the Southwest; Troy, MI, Warren, MI, Detroit, MI, and Kansas City, MO in the Midwest; and Pittsburgh, PA in the Northeast. Additional smaller counties—especially in ID, WI, and UT—also stand out for their high exemption rates.
- We analyzed the relationship between NME rates and actual vaccine coverage, and found an inverse association between NME rate and measles, mumps, and rubella (MMR) vaccine coverage of kindergarteners in these states (*P* = 0.03 by Spearman correlation), indicating that states with higher overall NME rates do in fact have lower MMR vaccine coverage (*P* = 0.007 by beta regression).
- Our findings indicate that new foci of antivaccine activities are being established in major metropolitan areas, rendering select cities vulnerable for vaccination-preventable diseases. As noted by the recent experience in Anaheim, California, low vaccination rates resulted in a measles outbreak. In contrast, state closure of NMEs has resulted in an increase of MMR coverage.

According to the 2015 National Immunization Survey, only 72.2% of children aged 19 to 35 months in the United States were fully vaccinated as per guidelines from the Advisory Committee on Immunization Practices [1]. Due to parental concerns about vaccine safety and efficacy, many families choose to opt out their children from vaccinations required for school entry by obtaining nonmedical exemptions (NMEs) based on religious or philosophical beliefs. In 2016, 18 states permitted NMEs due to philosophical beliefs [2]. A detailed analysis of NMEs within each of the 18 states reveals that several counties, including those with large metropolitan areas, are at high risk for vaccine-preventable pediatric infection epidemics.

## The rise in NMEs in 12 of 18 US states

NME data were collected from all 18 states currently permitting philosophical-belief NMEs (Arkansas [AR], Arizona [AZ], Colorado [CO], Idaho [ID], Louisiana [LA], Maine [ME], Michigan [MI], Minnesota [MN], Missouri [MO], North Dakota [ND], Ohio [OH], Oklahoma [OK], Oregon [OR], Pennsylvania [PA], Texas [TX], Utah [UT], Washington [WA], and Wisconsin [WI]). Of note, MO permits philosophical-belief exemptions only for child care facilities, but not public schools. Sixteen of the 18 states also allow religious exemptions, except LA and MN: “The existing statute in Minnesota and Louisiana does not explicitly recognize religion as a reason for claiming an exemption; however, as a practical matter, the non-medical exemption may encompass religious beliefs” [2]. VT, CA, MS, and WV were excluded from our analysis because they no longer have NMEs in their respective states. From our 18 included states, state-level data were collected from state health departments and/or the US Centers for Disease Control and Prevention (CDC) [3] and analyzed by school year from 2009–2010 to 2016–2017. The state NME rate is represented by the number of entering kindergarteners with a documented NME out of the total kindergarten enrollments in the state. Specifically, the data were obtained from CDC Morbidity and Mortality Weekly Report’s (MMWR) annual reports (AR, AZ, CO, ID, LA, ME, MI, MN, MO, ND, OH, OK, OR, PA, TX, UT, WA, and WI) and state health departments (AR, AZ, ID, LA, MI, MO, ND, OR, PA, and UT). CDC data from the 2010 to 2011 school year was not available for analysis; however, 10 of the 18 states individually provided data for the 2010 to 2011 school year. Because all data are represented as aggregated totals and not personally identifying, an exemption was obtained from the Baylor College of Medicine Institutional Review Board for this study.

**Fig 1:**
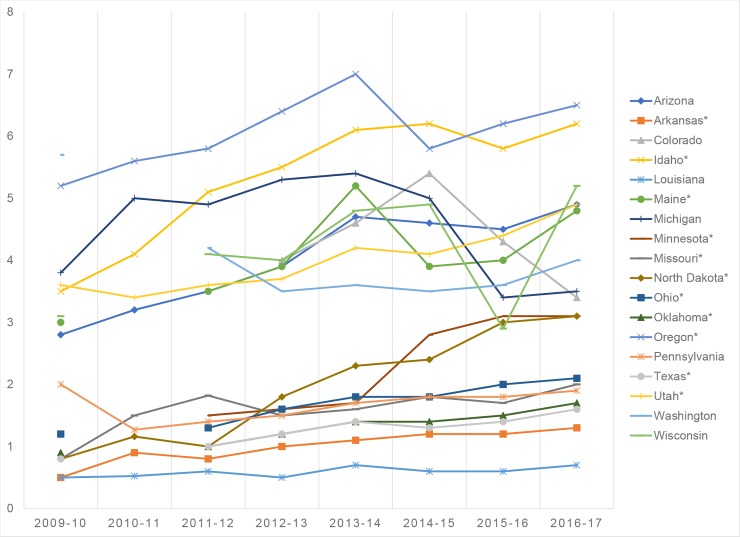
Increasing nationwide trend in kindergarten NME rates from 2009 to 2017. The asterisk (*) indicates states demonstrating an upward trend of kindergarteners with NMEs. NME, nonmedical exemption.

From our analysis, 12 of the 18 states permitting religious and philosophical-belief NMEs demonstrated an overall upward trend of enrolling kindergarteners with NMEs since 2009: AR, AZ, ID, ME, MN, MO, ND, OH, OK, OR, TX, and UT (Fig 1). All 12 states had a *P* < 0.05 by Mann-Kendall trend test. In general, the average rate of NMEs appears to have accelerated the most between the years 2009 and 2014, with some states exhibiting a plateau over the last 3 years. However, in states such as AR, ND, OH, OK, TX, UT, and others, the rates of NMEs continue to climb to the present day.

## Going granular: Vulnerable communities identified by high NME rates at the county level

Beyond the statewide data, many county-level NME rates were publicly available from state health departments for the school year 2016 to 2017. State- and county-level data included in our analysis have also been made publicly accessible (doi 10.6084/m9.figshare.6288968). County NME rate is defined as the number of enrolling kindergarteners with an NME out of the total kindergarten enrollments (public and private) in the county. Some data inconsistencies were present, including some missing county data for 3 states (AR, ND, OR), absent county data for 4 states (CO, LA, OH, and OK), and lacking data from the year 2016 to 2017 for 3 states (AR, PA, and TX). For AR, PA, and TX, we used data from the 2015 to 2016 school year. Missing data from NME-permitting states were either not collected by the state or publicly inaccessible.

**Fig 2:**
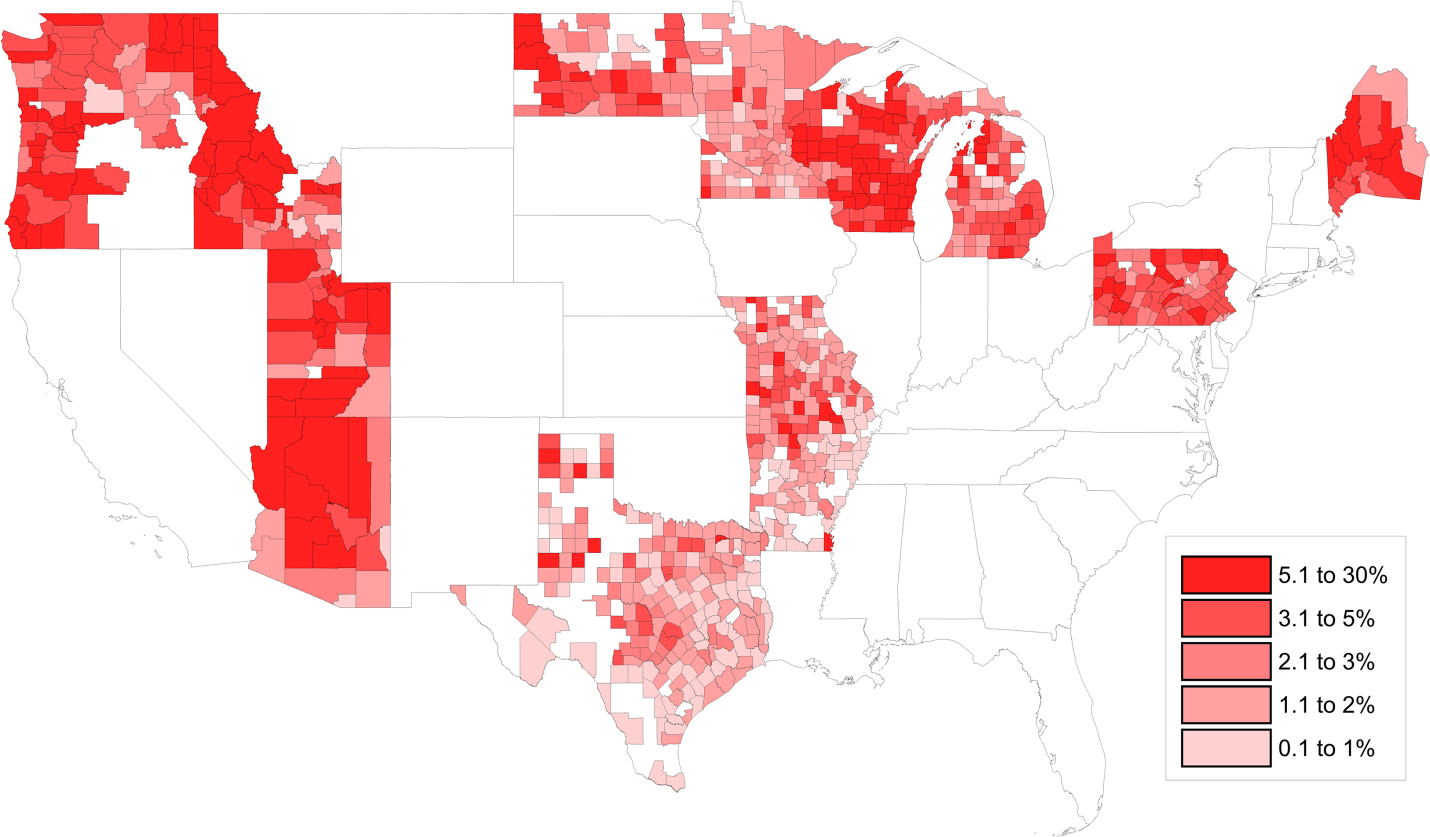
Heat map of county-level NME rates in 2016 to 2017. NME, nonmedical exemption.

As shown in Fig 2 and Table 1, of the 14 states that were analyzed, ID had an abundance of counties with the highest NME rates: Camas (26.67%), Bonner (19.65%), Valley (18.18%), Custer (17.14%), Idaho (16.06%), Boise (15.63%), Kootenai (14.91%), and Boundary (14.61%). WI’s Bayfield County was ranked 6th in overall NME percent (15.70%), and UT’s Morgan County (14.55%) was ranked 10th. Generally, the 10 counties with the highest NME rates in the country have fewer than 50,000 persons and are located in rural regions of the state.

**Table 1:** Ranking of the 10 leading counties by NME rates per kindergarten population.

Rank	County	State	Largest City by Population	NME Rate, 2016–2017
1	Camas	Idaho	Fairfield	26.67%
2	Bonner	Idaho	Sandpoint	19.65%
3	Valley	Idaho	McCall	18.18%
4	Custer	Idaho	Challis	17.14%
5	Idaho	Idaho	Grangeville	16.06%
6	Bayfield	Wisconsin	Washburn	15.70%
7	Boise	Idaho	Horseshoe Bend	15.63%
8	Kootenai	Idaho	Coeur d’Alene	14.91%
9	Boundary	Idaho	Bonners Ferry	14.61%
10	Morgan	Utah	Morgan	14.55%

Abbreviation: NME, nonmedical exemption.

**Fig 3:**
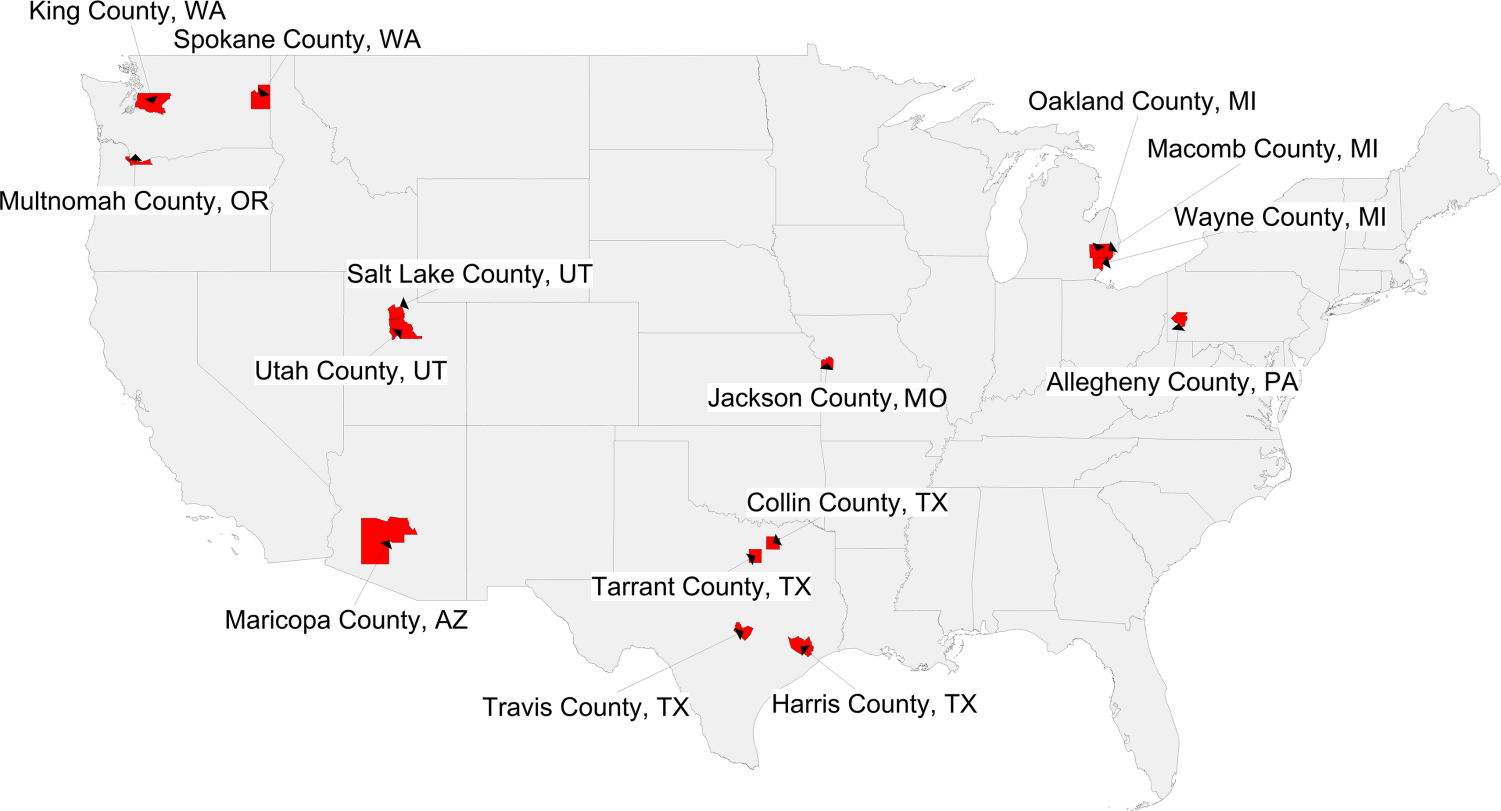
Heat map of counties with >400 kindergarteners with NMEs in 2016 to 2017. NME, nonmedical exemption.

Furthermore, we examined total numbers of kindergarteners with NMEs per county to identify focal areas with large numbers of potentially vulnerable pediatric populations. County NME totals were also provided by state health departments. The exception is MO, whose private kindergarten (2015–2016) and public kindergarten (2014–2015) enrollment numbers were taken together from the National Center for Education Statistics (nces.ed.gov) to derive NME raw counts. Shown in Fig 3 and Table 2 are the counties—associated with large metropolitan areas—where more than 400 kindergarteners have received NMEs. They include Phoenix, AZ (Maricopa County); Salt Lake City, UT and Provo, UT (Salt Lake and Utah Counties, respectively); Seattle, WA and Spokane, WA (King and Spokane Counties, respectively); Portland, OR (Multnomah County); Troy, MI, Warren, MI, and Detroit, MI (Oakland, Macomb, and Wayne Counties, respectively); Houston, TX, Fort Worth, TX, Plano, TX, and Austin, TX (Harris, Tarrant, Collin, and Travis Counties, respectively); Pittsburgh, PA (Allegheny County); and Kansas City, MO (Jackson County). The high numbers of NMEs in these densely populated urban centers suggest that outbreaks of vaccine-preventable diseases could either originate from or spread rapidly throughout these populations of unimmunized, unprotected children. The fact that the largest count of vaccine-exempt pediatric populations originate in large cities with busy international airports may further contribute to this risk.

**Table 2:** Ranking of the leading metropolitan areas with >400 total kindergarten NMEs.

Rank	County	State	Largest City by Population	NME Total, 2016–2017
1	Maricopa	Arizona	Phoenix	2,947
2	Salt Lake	Utah	Salt Lake City	956
3	King	Washington	Seattle	940
4	Multnomah	Oregon	Portland	711
5	Oakland	Michigan	Troy	686
6	Utah	Utah	Provo	662
7	Harris	Texas*	Houston	592
8	Tarrant	Texas*	Fort Worth	518
9	Collin	Texas*	Plano	478
10	Macomb	Michigan	Warren	477
11	Wayne	Michigan	Detroit	466
12	Allegheny	Pennsylvania*	Pittsburgh	424
13	Travis	Texas*	Austin	413
14	Jackson	Missouri	Kansas City	412
15	Spokane	Washington	Spokane	405

*Indicates data from 2015 to 2016.

Abbreviation: NME, nonmedical exemption.

## Are high NME rates associated with preventable and costly disease outbreaks?

**Fig 4:**
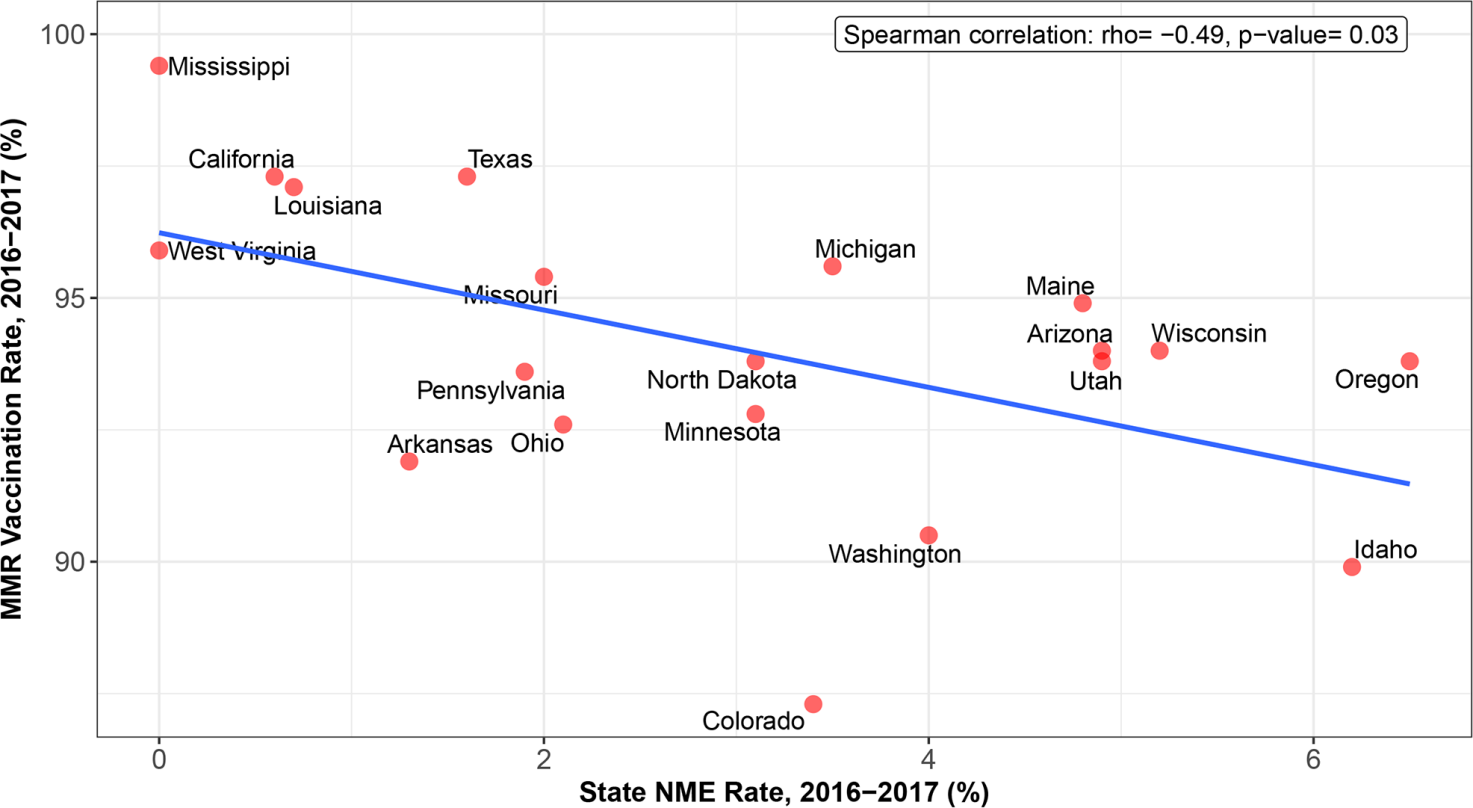
Negative relationship between state percentage of kindergarten MMR vaccine uptake and NME rate in the 2016 to 2017 school year. MMR, measles, mumps, and rubella; NME, nonmedical exemption.

Whereas measles was thought to have been eliminated from the US in 2000, we have seen local outbreaks of this vaccine-preventable disease and others, like measles and pertussis (whooping cough), in recent years due to inadequate immunization coverage in schools. For instance, a child with an NME from the measles, mumps, and rubella (MMR) vaccine is 35 times more likely to contract measles than is a vaccinated child [4]. Moreover, a child without the diphtheria, tetanus, and acellular pertussis (DTaP) vaccine is 3 times more likely to contract pertussis than is a vaccinated child [5]. NMEs weaken herd immunity that protects the population at large, particularly children who are unable to get vaccinated for medical reasons. The target vaccination coverage rate to achieve the ideal herd immunity is 90% to 95%, depending on the infectious agent [6]. To evaluate the influence of NMEs on vaccine uptake, Spearman correlation was calculated between state NME rate (%) and MMR vaccination rate (%) for 20 states either allowing NMEs (OK was excluded due to lack of MMR vaccine data) or 3 control states prohibiting NMEs in the 2016 to 2017 school year (CA, MS, WV) (CDC MMWR annual report) using R programming language (version 3.4.2) with the “cor.test” function. As shown in Fig 4 and Table 3, there was a significant inverse association between state NME rate and MMR vaccination rate by Spearman correlation (*P* = 0.03; Fig 4) and beta regression (*P* = 0.007). Similarly, we calculated Spearman correlation between state NME rate and MMR rate for all 50 US states and the District of Columbia. States with no information for either NME rate or MMR vaccination rate were excluded (CO, IL, MN, MO, OK, and WY). From this analysis, we found a significant inverse association between state NME rate and MMR vaccination rate (*P* = 0.04) as compared to states allowing NMEs. Overall, states with more NME students exhibited lower MMR vaccination rates. In contrast, states that have banned NMEs—MS, CA, and WV—exhibit the highest MMR vaccine uptake and lowest incidence of vaccine-preventable diseases.

**Table 3:** Numbers supporting the relationship. States that do not permit NMEs, included for comparison.

State	MMR Vaccination Rate, 2016–2017	NME Rate, 2016–2017	Kindergarten Enrollment
Arizona	94	4.9	83,627
Arkansas	91.9	1.3	39,666
California*	97.3	0.6	575,305
Colorado	87.3	3.4	64,440
Idaho	89.9	6.2	22,589
Louisiana	97.1	0.7	55,257
Maine	94.9	4.8	13,834
Michigan	95.6	3.5	118,777
Minnesota	92.8	3.1	69,140
Mississippi*	99.4	0	40,509
Missouri	95.4	2	73,355
North Dakota	93.8	3.1	9,799
Ohio	92.6	2.1	137,542
Oregon	93.8	6.5	52,184
Pennsylvania	93.6	1.9	143,888
Texas	97.3	1.6	389,999
Utah	93.8	4.9	49,073
Washington	90.5	4	87,142
West Virginia*	95.9	0	28,666
Wisconsin	94	5.2	67,607

*Indicates data from 2015 to 2016.

Abbreviation: MMR, measles, mumps, and rubella; NME, nonmedical exemption.

## Discussion

Our analysis reveals that, since 2009, NMEs have risen in 12 of the 18 states that currently allow philosophical-belief exemptions. A recent analysis found that the average annual change in state vaccine exemption rates in recent school years has begun to level off nationally, whereas it had been increasing in earlier years [7]. In at least one-half of the 18 states allowing NMEs, the rates have begun to plateau over the last few years. However, it is important to note that NMEs continue to rise in at least one-third of the 18 states, with no signs of slowing in those rates. In addition, in those states with plateauing levels, the potential for outbreaks still exists.

To better understand the problem of NMEs in the US, it is also helpful to examine the rates at the county level, given that NMEs are not distributed homogeneously within each state. Indeed, there are several counties representing hotspots of high NME rates, including some counties associated with large metropolitan areas where more than 400 kindergarten-aged children are not receiving their vaccines. For instance, TX has seen a particularly dramatic increase in NMEs of students in kindergarten through the 12th grade, multiplying nearly 20-fold since the policy’s inception in 2003 [6,8]. Our analysis identified the following hotspot metropolitan areas: Seattle, WA, Spokane, WA, and Portland, OR in the Northwest; Phoenix, AZ, Salt Lake City, UT, Provo, UT, Houston, TX, Fort Worth, TX, Plano, TX, and Austin, TX in the Southwest; Troy, MI, Warren, MI, Detroit, MI, and Kansas City, MO in the Midwest; and Pittsburgh, PA in the Northeast. It is important to note that our study is limited in its ability to correlate outbreaks with high NME communities, so the subsequent years will be critical in seeing how these potentials play out in a real-world setting.

Strict policy changes have elicited significant decreases in NME rates, with philosophical-belief NME prohibitions serving as an effective policy intervention. Several states have also attempted to decrease the NME rate through less stringent means (S1 Table). One common option is requiring parents to view an educational module prior to obtaining an NME (OR in 2014, MI in 2015), yet the effect of curbing the NME rate may not persist beyond the short term. This assertion is supported by an initial drop in OR’s rate followed by a steady climb since 2014. Furthermore, AR—which mandated parental education and an annual application for an NME in 2003—has nonetheless seen a yearly increase in NMEs. In 2011, WA enacted a more rigorous requirement of a physician’s signature prior to obtaining an NME. Although the rate declined in the following school year, the outcome was short-lived, and the rate plateaued in subsequent years. Another potentially influential policy was in the form of a federal grant issued by ME’s state government in 2014 to train healthcare providers on effective communication surrounding vaccines. However, it is unclear whether this multiyear grant was primarily responsible for reversing the NME trend in a single year. In contrast to the states that sought to decrease NMEs, PA controversially began allowing the philosophical-beliefs exemption in 2014.

The measles epidemic from 2014 to 2015 originating from the Disneyland theme park in Anaheim, CA was a consequence of low MMR vaccination coverage of children (50%–86% vaccination rate among the exposed population) [9]. In direct response to the measles epidemic, the CA State Legislature passed Senate Bill 277 (SB 277) [10], which placed a statewide ban on NMEs starting January 1, 2016 to mirror previous actions in MI and WV [11]. The policy’s effect has been both immediate and significant. The number of kindergarteners with NMEs from 2016 to 2017 dropped to 3,133, the lowest that the state has seen in over a decade [11]. Each county reported significantly fewer children with NMEs. Accordingly, the new law also increased the state’s kindergarten vaccination rates, which reached a record high during 2016 to 2017: 95.6% of kindergarteners received all required vaccines compared to 92.8% in 2015 to 2016 [11]. Given the effectiveness of closing the exemption policy, evidence suggests that other states should consider discontinuing the NME option and protect schoolchildren from vaccine-preventable diseases.

Stricter legislative action to close NMEs should become a higher priority because of the positive correlation between leniency of state vaccination policies and exemption rates. On average, states with policies that permit both types of NMEs have 2.5 times higher exemption rates [12]. Although many states demonstrate rising exemption totals irrespective of the exemption policy, states that make it harder to obtain exemptions have demonstrated slower-growing opt-out rates over time. Therefore, it is critical to identify states at highest risk for an outbreak due to a rise in NMEs.

As has been demonstrated by past policy changes, uptake in vaccination rates by communities requires multiple measures (S1 Table). It has been pointed out that a vertical policy change, such as the one highlighted above, should be accompanied by expanded horizontal efforts to increase access to vaccines and booster shots, educational programs, and awareness campaigns [12–14]. For instance, healthcare providers might play a greater role in promoting vaccination. The presumptive strategy of health communication, which promotes the safety and routineness of vaccination, between provider and parent has been shown to discourage parents from seeking to obtain NMEs [13]. Furthermore, greater community involvement through churches, neighborhood clinics, and provaccination celebrity allies would help to reach both undervaccinated communities—who are affected by vaccine access-related issues—and unvaccinated groups. Recently, Opel and colleagues identified key policies in the areas of enforcement and reimbursement for pediatricians or other providers who advise vaccine-hesitant parents [14]. As indicated by the increase in NME rates despite ND’s policy passage to increase vaccine supply without physician education (S1 Table), it is critical to achieve community buy-in through targeted education campaigns. A multifaceted approach with a central emphasis on discontinuing NMEs will work to increase vaccination rates across a wider scope of the American population and reduce the risk of otherwise preventable and costly disease outbreaks.

The results reported here for the US have potential relevance internationally. While NMEs continue to rise in most of the 18 US states that allow them, several European countries, including France and Italy, as well as Australia, have taken measures to either make vaccines compulsory or even fine parents who refuse to vaccinate their children [15–17]. Romania has experienced serious and large measles outbreaks and may also tighten vaccine legislation [18]. Our concern is that the rising NMEs linked to the antivaccine movement in the US will stimulate other countries to follow a similar path. It would be especially worrisome if the very large low- and middle-income countries—such as Brazil, Russia, India, and China (the BRIC nations), or Bangladesh, Indonesia, Nigeria, and Pakistan—reduce their vaccine coverage. In such a case, we could experience massive epidemics of childhood infections that may threaten achievement of United Nations global goals [19].

## Supporting information

S1 Table State policies influencing NME rates. NME, nonmedical exemption.
(DOCX)

## Abbreviations

BRIC: Brazil, Russia, India, and China
CDC: Centers for Disease Control and Prevention
DTaP: diphtheria, tetanus, and acellular pertussis
MMR: measles, mumps, and rubella
MMWR: Morbidity and Mortality Weekly Report
NME: nonmedical exemption
