# Correction: The state of the antivaccine movement in the United States: A focused examination of nonmedical exemptions in states and counties

**DOI:** 10.1371/journal.pmed.1002616

**Published:** 2018-07-06

**Authors:** Jacqueline K. Olive, Peter J. Hotez, Ashish Damania, Melissa S. Nolan

After publication of this article, the authors became aware of an error in the quoted number of philosophical-belief vaccine nonmedical exemptions (NMEs) in Allegheny County, Pennsylvania. The quoted number of 424 exemptions resulted from an inadvertent doubling of the correct number of 212. The authors apologize for this error. All the data and analyses in the paper have now been thoroughly checked, and we can verify that, while the error affected all entries in the Pennsylvania county-level dataset, no data from other states were affected. Therefore, the authors would like to correct the text and figures of the article as follows, to remove Allegheny County from the maps and rankings of areas with >400 kindergarteners with NMEs in 2016-2017. In addition to Allegheny County, NME rates for all counties in Pennsylvania have been corrected in Fig 2.

There were also two minor errors in presentation that need to be corrected, regarding the list of states in the second summary point and an asterisk missing from Fig 1.

**Fig 1:**
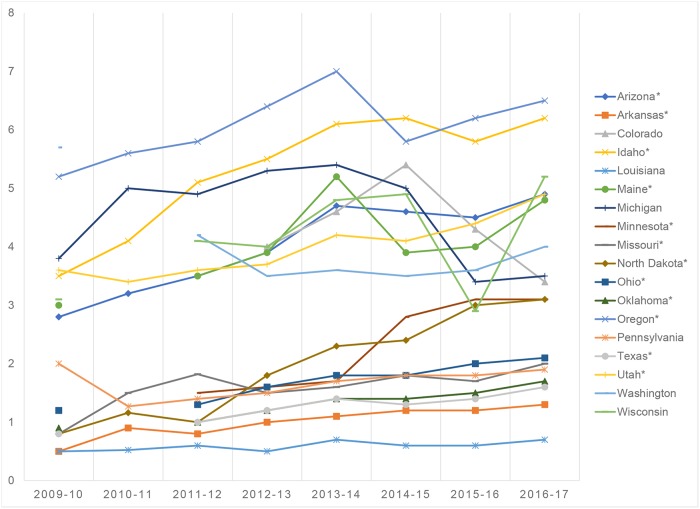
Increasing nationwide trend in kindergarten NME rates from 2009 to 2017. The asterisk (*) indicates states demonstrating an upward trend of kindergarteners with NMEs. NME, nonmedical exemption.

The first sentence of the second Summary point should read “Since 2009, the number of philosophical-belief vaccine nonmedical exemptions (NMEs) has risen in 12 of the 18 states that currently allow this policy: Arizona (AZ), Arkansas (AR), Idaho (ID), Maine (ME), Minnesota (MN), Missouri (MO), North Dakota (ND), Ohio (OH), Oklahoma (OK), Oregon (OR), Texas (TX), and Utah (UT).”

We would also like to amend the third Summary point, to substitute the names of the counties from which data were derived for the names of cities within those counties. The first sentence of the third Summary point should read “Several US “hotspot” metropolitan areas stand out for their very large numbers of NMEs. They include King County, WA, Spokane County, WA, and Multnomah County, OR, in the Northwest; Maricopa County, AZ, Salt Lake County, UT, Utah County, UT, Harris County, TX, Tarrant County, TX, Collin County, TX, and Travis County, TX, in the Southwest; and Oakland County, MI, Macomb County, MI, Wayne County, MI, and Jackson County, MO, in the Midwest.”

Fig 1 has been corrected to add an asterisk next to Arizona.

Fig 2 now shows corrected NME rates for all counties in Pennsylvania. Pennsylvania’s county level NME rates have been verified with the original source, provided by the Pennsylvania Department of Health, to ensure accuracy.

**Fig 2:**
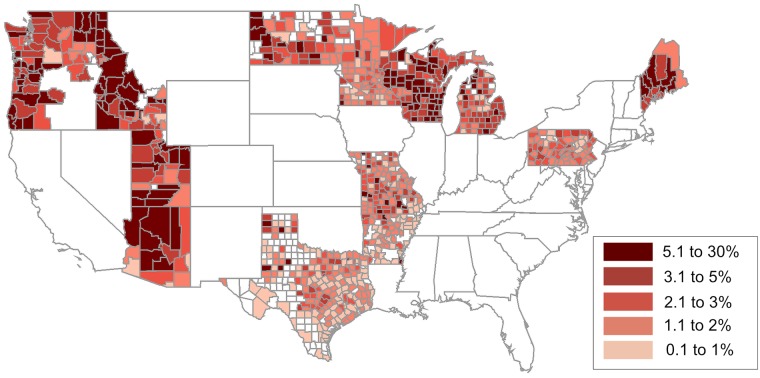
Heat map of county-level NME rates in 2016 to 2017. NME, nonmedical exemption.

The third paragraph of the section subtitled “Going granular” should read “Furthermore, we examined total numbers of kindergarteners with NMEs per county to identify focal areas with large numbers of potentially vulnerable pediatric populations. County NME totals were also provided by state health departments. The exception is MO, whose private kindergarten (2015–2016) and public kindergarten (2014–2015) enrollment numbers were taken together from the National Center for Education Statistics (nces.ed.gov) to derive NME raw counts. Shown in Fig 3 and Table 2 are the counties—associated with large metropolitan areas—where more than 400 kindergarteners have received NMEs. They include Phoenix, AZ (Maricopa County); Salt Lake City, UT and Provo, UT (Salt Lake and Utah Counties, respectively); Seattle, WA and Spokane, WA (King and Spokane Counties, respectively); Portland, OR (Multnomah County); Troy, MI, Warren, MI, and Detroit, MI (Oakland, Macomb, and Wayne Counties, respectively); Houston, TX, Fort Worth, TX, Plano, TX, and Austin, TX (Harris, Tarrant, Collin, and Travis Counties, respectively); and Kansas City, MO (Jackson County). The high numbers of NMEs in these densely populated urban centers suggest that outbreaks of vaccine-preventable diseases could either originate from or spread rapidly throughout these populations of unimmunized, unprotected children. The fact that the largest count of vaccine-exempt pediatric populations originate in large cities with busy international airports may further contribute to this risk.”

**Fig 3:**
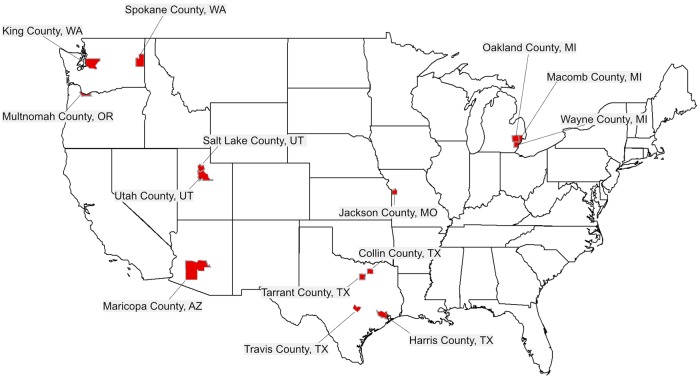
Heat map of counties with >400 kindergarteners with NMEs in 2016 to 2017. NME, nonmedical exemption.

**Table 2:** Ranking of the leading metropolitan areas with >400 total kindergarten NMEs.

Rank	County	State	Largest City by Population	NME Total, 2016–2017
1	Maricopa	Arizona	Phoenix	2,947
2	Salt Lake	Utah	Salt Lake City	956
3	King	Washington	Seattle	940
4	Multnomah	Oregon	Portland	711
5	Oakland	Michigan	Troy	686
6	Utah	Utah	Provo	662
7	Harris	Texas*	Houston	592
8	Tarrant	Texas*	Fort Worth	518
9	Collin	Texas*	Plano	478
10	Macomb	Michigan	Warren	477
11	Wayne	Michigan	Detroit	466
12	Travis	Texas*	Austin	413
13	Jackson	Missouri	Kansas City	412
14	Spokane	Washington	Spokane	405

*Indicates data from 2015 to 2016.

Abbreviation: NME, nonmedical exemption.

Fig 3 has been corrected to remove Allegheny County, Pennsylvania.

In Table 2, the county and state ranked #12 (Allegheny, Pennsylvania, Pittsburgh) has been removed, and the subsequent counties have been moved up in ranking to be #12-Travis County, Texas, #13-Jackson County, Missouri, and #14-Spokane County, Washington.

The penultimate line in the second paragraph of the Discussion should be “Our analysis identified the following hotspot metropolitan areas: Seattle, WA, Spokane, WA, and Portland, OR in the Northwest; Phoenix, AZ, Salt Lake City, UT, Provo, UT, Houston, TX, Fort Worth, TX, Plano, TX, and Austin, TX in the Southwest; Troy, MI, Warren, MI, Detroit, MI, and Kansas City, MO in the Midwest.”

The Acknowledgments should read: We would like to acknowledge the following state health departments that provided us with data wherever possible: AR Department of Health, ID Department of Health and Welfare, LA Department of Health, MO Department of Health and Senior Services, ND Department of Health, OR Health Authority, TX Department of State Health Services, UT Department of Health, and WI Department of Health Services.
