# Supplementary material for: The state of the antivaccine movement in the United States: A focused examination of nonmedical exemptions in states and counties
Source: PLoS Med. 2018 Jun 12;15(6):e1002578. doi: 10.1371/journal.pmed.1002578 (PMC5997312; doi:10.1371/journal.pmed.1002578)
Supplement: S1 Table — NME, nonmedical exemption. (DOCX) [file pmed.1002578.s001.docx]

**S1 Table. State policies influencing NME rates.**

| State | Policy Change(s), 2009-2016 | Year |
| --- | --- | --- |
| California | SB 277: prohibits NMEs from vaccination requirements of public and private schools 🡪 **Decrease in NMEs** | 2016 |
| Colorado | HB-1288: schools/childcares must make vaccination and exemption rates publicly available upon request and online education module created 🡪 **Decrease in NMEs**; 25-4-902 C.R.S.: requires annual application for vaccine exemption | 2014; 2016 |
| Maine | Five-year federal grant that trained younger providers on how to talk to families about vaccines 🡪 **Decrease in NMEs** | 2014 |
| Michigan | R 325.176 (update to MDHHS Communicable and Related Diseases Administrative Rules): parents must obtain exemption waivers at county health department, sit through education session, and sign a form before obtaining NMEs 🡪 **Decrease in NMEs** | 2015 |
| North Dakota | SB 2276: universal state-supplied vaccines to increase vaccination rates🡪 **Increase in NMEs** | 2011 |
| Oregon | ORS 433.267: parents must watch an educational video online or get an education certificate at a doctor's office before obtaining NMEs 🡪 **Decrease in NMEs** | 2014 |
| Pennsylvania | 28 Pa. Code § 23-83, 84: permits personal beliefs exemption 🡪 **Increase in NMEs** | 2014 |
| Washington | Wash. Rev. Code Ann. § 28A.210.080, 90: requires health care practitioner signature before obtaining NMEs 🡪 **Decrease in NMEs** | 2011 |

*Sources:

- CA: <https://leginfo.legislature.ca.gov/faces/billNavClient.xhtml?bill_id=201520160SB277>
- CO: <https://www.colorado.gov/pacific/cdphe/house-bill-14-1288>, <https://leg.colorado.gov/sites/default/files/documents/2016a/bills/sl/2016A_sl_308.pdf>
- ME: <http://bangordailynews.com/2016/08/15/mainefocus/maine-got-millions-to-help-moms-and-babies-but-has-little-to-show-for-it/?ref=series>, <http://www.vaxmainekids.org/>
- MI: <http://www.michigan.gov/documents/mdch/2007-027_CH__Communicable_and_Related_Diseases_8-10-09__nonstrike_bold__298507_7.pdf>
- ND: <https://www.ndhealth.gov/Testimony/docs/4.pdf>
- OR: <https://www.oregonlaws.org/ors/433.267>
- PA: <http://www.pacode.com/secure/data/028/chapter23/s23.83.html>

WA: <http://apps.leg.wa.gov/Rcw/default.aspx?cite=28A.210.090>
